# Vaccinia Virus Activation and Antagonism of Cytosolic DNA Sensing

**DOI:** 10.3389/fimmu.2020.568412

**Published:** 2020-10-01

**Authors:** Misbah El-Jesr, Muad Teir, Carlos Maluquer de Motes

**Affiliations:** Department of Microbial Sciences, University of Surrey, Guildford, United Kingdom

**Keywords:** STING, CGAS, interferons, antiviral signaling, vaccinia virus (VACV)

## Abstract

Cells express multiple molecules aimed at detecting incoming virus and infection. Recognition of virus infection leads to the production of cytokines, chemokines and restriction factors that limit virus replication and activate an adaptive immune response offering long-term protection. Recognition of cytosolic DNA has become a central immune sensing mechanism involved in infection, autoinflammation, and cancer immunotherapy. Vaccinia virus (VACV) is the prototypic member of the family Poxviridae and the vaccine used to eradicate smallpox. VACV harbors enormous potential as a vaccine vector and several attenuated strains are currently being developed against infectious diseases. In addition, VACV has emerged as a popular oncolytic agent due to its cytotoxic capacity even in hypoxic environments. As a poxvirus, VACV is an unusual virus that replicates its large DNA genome exclusively in the cytoplasm of infected cells. Despite producing large amounts of cytosolic DNA, VACV efficiently suppresses the subsequent innate immune response by deploying an arsenal of proteins with capacity to disable host antiviral signaling, some of which specifically target cytosolic DNA sensing pathways. Some of these strategies are conserved amongst orthopoxviruses, whereas others are seemingly unique to VACV. In this review we provide an overview of the VACV replicative cycle and discuss the recent advances on our understanding of how VACV induces and antagonizes innate immune activation via cytosolic DNA sensing pathways. The implications of these findings in the rational design of vaccines and oncolytics based on VACV are also discussed.

## Introduction

Vaccinia virus (VACV) is the prototypic and the most widely and intensely studied member of the family Poxviridae, a family of cytoplasmic-replicating viruses harboring large ~200 kbp linear dsDNA genomes. VACV is a very immunogenic virus that served as a vaccine for the eradication of smallpox, the only human disease eradicated so far. The virus causing smallpox, variola virus (VARV), and VACV belong to the genus Orthopoxvirus (OPXV), which also includes several other viral species infecting mammals. The origins and evolution of VACV are complex, but likely to share an ancestor with the now extinct horsepox virus (1). Multiple VACV strains exist and were used during the smallpox vaccination campaign around the globe. Although highly similar, the genetic content of these strains may vary, possibly as a reflection of their historical passage and use (2). In 1990, the group of Enzo Paoletti published the genome sequence of VACV strain Copenhagen (COP), one of the most studied strains (3). In this review, the COP nomenclature is used to identify VACV genes, but reference is also given to the Western Reserve (WR) strain, which is commonly used as a research tool. Despite the huge potential of using VACV as a vaccine vector for other infectious diseases, the smallpox eradication campaign revealed side-effects and complications derived from the use of live replication-competent VACV strains, and highlighted the need for safer vaccines with safety records conforming with current standards. This need fuelled (i) the search for proteins contributing to virulence, and hence likely to attenuate the virus when removed, and (ii) the use of severely attenuated strains generally obtained after serial passages in cell culture. One of this highly attenuated strains is Modified Vaccinia virus Ankara (MVA), a derivative of the Turkish smallpox vaccine strain chorioallantoic VACV Ankara (CVA) that has lost many immunomodulatory and host range genes and is unable to replicate in human cells. The excellent safety profile of MVA and its rapid induction of immune protective responses has fostered its development as vaccine vector against multiple diseases (4). In addition, MVA has become a great tool to understand how VACV is sensed by the host innate immune system, thereby allowing rational improvement of vaccine design.

As a poxvirus VACV is a very complex virus. It follows an exclusively cytosolic replicative cycle, as opposed to most DNA viruses which replicate in the nucleus. Viral genome replication and assembly of nascent virions takes place in specific areas of the cytoplasm generally known as viral factories (5, 6). Establishment of viral factories is preceded by genome release and expression of early genes, which occurs inside the viral cores before these are dismantled and the genome becomes permissive for replication (7–10). Once produced nascent virions travel through the Golgi apparatus and mature into a double-membraned form known as extracellular virus (EV). EV mediate viral spread to neighboring cells and are critical to establish infection within an individual (11, 12). As the infection progresses, single-membraned virions known as mature virus (MV) accumulate inside the cytosol and are released upon cell lysis. In the host, VACV infection initiates in skin fibroblasts and proceeds to inflammatory monocytes recruited to the site of infection (13, 14), which can contain infection but also spread the virus through the blood stream. A core set of genes conserved amongst OPXV can be identified in the central part of the linear genome and are mostly involved in viral replication and morphogenesis. These conserved genes render OPXV antigenically similar and generate cross-protection after immunization. On the contrary, the genome termini are rich in accessory genes whose function is to modulate the host immune response and determine host range. Most of these genes are therefore specific to each member of the genus and have sometimes followed clear duplication and speciation events. For instance, VACV is rich in genes coding for proteins that resemble the cellular B-cell lymphoma (Bcl)-2 family despite having minimal sequence conservation amongst them (15, 16). Equally, other genus members such as cowpox virus (CPXV) or ectromelia virus (ECTV) are rich in genes coding for proteins containing Ankyrin repeats (17–19). Research on the functions of these proteins indicates strong convergent evolution on suppression of host innate immunity. Most of the VACV immunomodulatory genes are under the control of early promoters and are therefore deployed as soon as infection initiates. Some have been identified into the viral particle and may become immediately available upon entry (20–22). Between one-third and one-half of VACV proteins are estimated to interfere with the host immune response, some by more than one mechanism. Given this arsenal of immunomodulatory proteins it remains puzzling how VACV is such an immunogenic virus and induces potent humoral and cellular responses to self and foreign antigens. A unique property of VACV and poxviruses is to replicate in the cytosol, where most innate pattern recognition receptors (PRR) reside (23, 24). The innate immune system provides a rapid and robust response to invading pathogens that is well-known to impact and shape the subsequent adaptive response clearing the infection. The evolutionary interplay between host innate sensors and viral antagonists in the highly hostile cytosolic niche occupied by VACV is likely to profoundly determine the outcome of infection and therapeutic treatment.

Here, we review the current knowledge on how cells sense VACV infection through its DNA genome and how VACV in turn prevents this recognition. VACV produces several intracellular proteins targeting the core components of host DNA sensing signaling and others targeting components acting downstream. VACV also encodes soluble decoy receptors neutralizing some of the host cytokines induced by DNA sensing pathways such as interferons (IFN) and tumor necrosis factor (TNF)-α, but these are not covered here and we refer the reader to previous reviews on the topic (16, 25–27). Finally, we discuss the implications of VACV DNA sensing in the therapeutic use of VACV as a vaccine vector and oncolytic agent.

## Overview of Antiviral Cytosolic DNA Sensing

**Figure 1:**
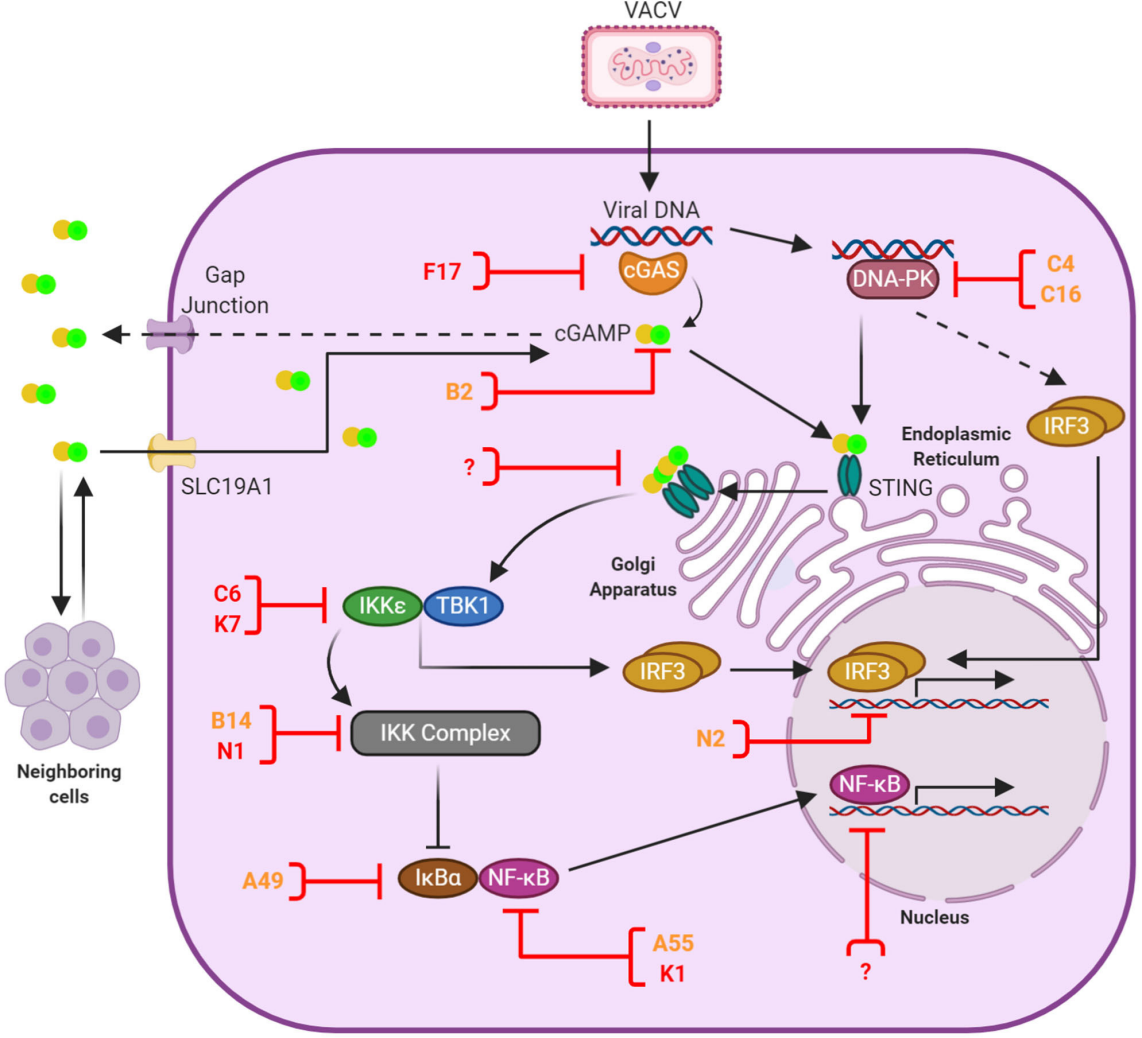
Anti-viral DNA sensing and its antagonism by VACV. Upon infection the VACV genome is released into the cytosol and recognized primarily by cGAS, although other sensors such as DNA-PK play a role in a cell-specific manner. Activated cGAS catalizes the production of 2′ 3′ -cGAMP, which binds and activates STING. In addition, cGAMP is transferred to neighboring cells via gap junctions and imported from the extracellular environment via the transporter SLC19A1. cGAMP-bound STING oligomers mediate the recruitment of TBK1, which subsequently leads to the activation of IRF3 and NF-κB signal transduction and the induction of anti-viral responses. A number of VACV proteins have the capacity to block cytosolic DNA sensing and are shown here in orange or red depending on the level of conservation (see Table 1). These proteins counteract multiple stages of the signaling cascade (shown here with a blocked line). Evidence exists for additional inhibitors acting in the cell nucleus, although they remain yet unidentified. At present no VACV inhibitors of STING have been discovered, but this is a convergent signaling point known to be blocked by several viruses. These potential target sites are indicated with a question mark.

The presence of foreign RNA and DNA within the cell cytosol is a clear sign of danger. Intracellular DNA is detected by a number of PRR that lead to a robust cellular response characterized by a rapid production of chemokines and cytokines including type I IFN (IFN-I) and the subsequent expression of IFN-stimulated genes (ISG). Induction of this antiviral response mostly relies on transcriptional activation by IFN responsive factors (IRF) and the nuclear factor κ-light-chain-enhancer of activated B cells (NF-κB), although transcription-independent mechanisms exist. This is the case of the Absent in Melanoma (AIM)-2-like receptors, which initiate the release of the potent inflammatory cytokines interleukin (IL)-1β and IL-18 upon recognition of cytosolic DNA (28, 29). Activation of antiviral IRF and NF-κB signaling in response to DNA mostly derive from cytosolic DNA sensors, but also from the membrane-bound Toll-like receptor (TLR)-9 which recognizes DNA contained in endosomal vesicles (30, 31). TLR9 expression is mostly restricted to specialized immune cells and it transduces signal via myeloid differentiation primary response 88 (MyD88) to eventually phosphorylate IRF3 and IRF7 (31, 32). Amongst cytosolic DNA sensors cyclic GMP-AMP synthase (cGAS) stands out as a critical molecule since it appears essential for IFN production in every setting where this has been tested (Figure 1). cGAS is a DNA-binding enzyme that produces the small second messenger 2′ 3′ -cyclic GMP-AMP (cGAMP) upon recognition of dsDNA (48–52). cGAS belongs to the family of nucleotydiltransferases, loosely related to the oligoadenylates synthetases (53). The unique phosphodiester linkage in cGAMP (the 2'-OH of GMP binds to the 5′ of AMP and the 3′ -OH of AMP binds to the 5′ phosphate of GMP) confers greater affinity to the stimulator of IFN genes (STING) than other reported cyclic dinucleotides (CDNs) (51, 54, 55). cGAS patrols the cell cytosol as a sensor for abnormal situations revealed by mislocalised DNA either from invading pathogens or cellular stress (56). Its product cGAMP binds to and activates STING to promote IRF and NF-κB activation in the stimulated cell (57–60), but also in unstimulated neighbor cells via intercellular and extracellular transfer (61–64).

Upon cGAMP binding, STING dimers translocate from the endoplasmic reticulum (ER) to Golgi compartments where several post-translational modifications, including palmitoylation and ubiquitylation, take place to establish the STING signalosome (65–68). Here the C-terminal tail of STING interacts with TANK-binding kinase (TBK)-1 to mediate IRF3 activation in a complex trans-phosphorylation process that has recently been enlightened by structural advances (69–71) and has been reviewed in detail elsewhere (72, 73). Phosphorylated IRF3 dimerises and translocates to the cell nucleus to drive expression of IRF-dependent genes including the IFNβ enhanceosome. It is also known that STING oligomerisation induces the subsequent degradation of the inhibitor of κB (IκB)-α and activate, albeit to a lower extent, NF-κB responses including the production of TNFα, IL-1β and IL-6 (57, 58, 74–76). Canonical NF-κB signaling deriving from cytokine receptors or TLR requires the use of TNF receptor-associated factor (TRAF)-6 or TRAF2 to activate the IκBα kinase (IKK) complex, which in turn phosphorylates IκBα and triggers its proteasomal degradation via the E3 ligase β-TrCP. STING responses require the IKK complex, which can be redundantly activated by TBK1 and its homolog IKKε in a manner that may involve the upstream kinase transforming growth factor-β-activated kinase 1 (TAK1) (76, 77). NF-κB would usually be found as the p65/p50 (RelA/NF-κB1) heterodimer, the most common of NF-κB protein dimers (78). Following the degradation of IκBα, NF-κB becomes free to move from the cytoplasm to the nucleus (78, 79). There it can induce the transcription of several target genes, which include immunomodulatory proteins as well as IκBα, which is required to maintain a negative feedback loop (80). There also exists a non-canonical pathway, which comes into play during DNA damage. This pathway involves the activity of ataxia telangiectasia mutated (ATM) and poly ADP-ribose polymerase 1 (PARP-1), initiating an outside signal from within the cell that goes through IFNγ-inducible protein 16 (IFI16) and STING, without the aid of cGAS, and eventually reaches the IKK complex and activates NF-κB (75).

**Table 1:** VACV proteins counteracting intracellular DNA sensing pathways.

**Protein**	**Size (kDa)**	**Conservation^**a**^**	**Function**	**Virulence factor**	**Key references**
A49	18.7	Most OPXV	Targets β-TrCP; NF-κB inhibitor	Yes	(33, 34)
A55	64.6	Most OPXV	NF-κB inhibitor; prevents NF-κB nuclear translocation	Yes	(35)
B2/Poxin	24.6	VACV; vSchlafen in OPXV	2′,3′-cGAMP nuclease	Yes	(36)
B14	17.3	Most OPXV	NF-κB inhibitor; targets IKKβ	Yes	(37, 38)
C4	37.2	Most OPXV	DNA-PK inhibitor; binds to Ku proteins	Yes	(39)
C6	17.3	OPXV	IRF3/7 inhibitor; targets TBK1 adaptors	Yes	(40)
C16	37.5	Most OPXV	DNA-PK inhibitor; binds to Ku protein	Yes	(39, 41)
F17	11.3	OPXV	Dysregulates mTOR; downregulates cGAS and STING	Essential for growth	(42)
K1	32.4	OPXV	NF-κB inhibitor; prevents IκBα degradation	Yes	(43)
K7	17.4	OPXV	IRF3 inhibitor; targets DDX3	Yes	(44)
N1	13.9	OPXV	NF-κB inhibitor	Yes	(37, 45, 46)
N2	20.8	Most OPXV	Nuclear IRF3 inhibitor	Yes	(47)

a *Conservation within the OPXV is indicated*.

Before and after the discovery of cGAS other molecules were found to affect DNA sensing signaling. Although the specific biochemical pathways used by these molecules are not always deciphered, nearly all of them converge on STING and may act as cofactors, modulators or regulators of the cGAS-cGAMP-STING axis in a species, cell type and/or self- vs. non-self-specific manner. This list includes DNA-dependent activator of interferon (DAI) (81), RNA polymerase III (82, 83), IFI16 (84), the DExD/H-box helicases DHX9, DDX36 (85) and DDX41 (86); the DNA damage proteins Ku70/80 (87), DNA-PK (88), Mre11 (89) and PQBP1 (90); LSM14A (91); and G3BP1 (92). For a detailed review of this topic, we refer the reader to recent publications (72, 73). Two of these molecules, IFI16 and DNA-PK, have been reported to have direct roles in VACV sensing and are discussed below in more detail. One of them, namely DNA-PK, has been recently implicated in STING-independent IFN-I production in response to DNA and involves the downstream signaling of the heat shock protein HSPA8, in what is being referred to as the STING-independent DNA sensing pathway (SIDSP) (93).

## DNA Sensing Activation in Response to VACV

Understanding the interplay between host DNA sensing and VACV is difficult because VACV suppresses cell responses through expression of multiple antagonists acting upstream and downstream of these pathways (16, 94). Insights are therefore usually gained using attenuated VACV strains such as MVA that have lost most of these antagonists or employing live animal models that can reproduce the complex immunological responses generated upon infection. VACV can cause pathology in commonly used mouse breeds such as Balb/c or C57BL/6, so genetically engineered mice lacking specific DNA sensing and innate immune molecules can provide insights into VACV immunity, with the caveat that DNA sensing in mice may differ from humans as it is becoming increasingly reported (93, 95). Inoculation of VACV into transgenic mice deficient for cGAS revealed a mild increase in viral replication and tissue pathology (14, 96) that correlated with reduced IFN-I expression, which was also observed in STING deficient mice (14). Similar experiments performed with ECTV, a mouse-specific virus and the causative agent of mousepox, showed enhanced mortality in STING deficient mice (97), but varied susceptibility in cGAS deficient mice (97, 98). In contrast, mice lacking IFI204 (the mouse ortholog of IFI16) survived ECTV infection and did not exhibit significant differences in viral replication and viral burden (98). Whilst these studies demonstrated the role of cGAS and STING in mounting responses against VACV and ECTV *in vivo*, the observed effects were rather mild. This suggests that VACV and ECTV minimize the activity of this pathway to the point that genetic ablation of an already targeted signaling nodule had reduced effect on the overall response. The discovery of viral inhibitors of DNA sensing (discussed below) has allowed a better assessment of the impact of the cGAS-cGAMP-STING axis and recently, ECTV lacking the viral cGAMP nuclease vSchlafen has shown extreme attenuation in multiple models of infection (99). This has revealed the enormous importance of cGAS-STING in poxvirus immunity.

The use of the highly attenuated VACV strain MVA has proven pivotal in understanding DNA sensing immunity against VACV. The MVA genome is significantly shorter than its parental CVA due to five large deletions and additional mutations in at least 25 genes resulting in truncated proteins (100). Since many of these proteins are non-essential immune modulators, it is not surprising that MVA acts as potent inducer of immune responses in most mammalian cells. Before the discovery of DNA sensing pathways, MVA infection was known to be recognized by TLR dependent and independent pathways including the RNA sensor melanoma differentiation-associated protein (MDA)-5 in macrophages and dendritic cells (101–104). MDA5 converges onto IRF3 signaling and IFN-I production via mitochondrial antiviral signaling protein (MAVS) and recognizes long dsRNA in the cytosol (105, 106), a by-product of poxviral gene expression in this compartment. Subsequently, several studies have shown that IFN-I induction by MVA relies on cGAS and its downstream adaptor STING (88, 94, 107, 108). Deletion or depletion of cGAS or STING in mouse and human cells results in impaired induction of IFNα, IFNβ, and CXCL10 in response to MVA. These findings are in line with results obtained with other poxviruses and in the context of replicative VACV lacking specific DNA sensing inhibitors (97, 109, 110). In addition to cGAS other DNA sensors have been implicated in VACV immunity: DNA-PK and IFI16. DNA-PK consists of a heterodimer of the DNA-binding proteins Ku70 and Ku80 and DNA-PKcs, its catalytic subunit. DNA-PK is best known for its role in DNA repair and V(D)J recombination (111), but it also acts as a PRR in response to intracellular DNA (88). DNA-PK leads to IRF3 activation and cytokine production including IFN-I in response to cytoplasmic DNA, particularly in fibroblasts. Although the specific biochemical pathway induced by DNA-PK is not as clear as for cGAS, it has been shown to trigger innate immune activation through the cGAS-STING-IRF3 pathway (88, 112) and more recently, in a STING-independent manner (93). DNA-PK is essential for cell viability and this limits its genetic manipulation. Nonetheless, murine fibroblasts deficient for specific DNA-PK components show reduced levels of IFN-I, CXCL10 and IL-6 when infected with MVA whilst retaining complete responsiveness to RNA virus infection, and this correlated with DNA-PK translocation to viral factories (88). IFI16 has also been shown to translocate to viral factories, particularly in keratinocytes, and this correlated with reduced CCL5 and ISGs production in response to MVA (113), although no difference in IFN-I has been observed in macrophages (107), suggesting a cell-specific role for IFI16 in sensing VACV infection. IFI16 is a critical sensor for nuclear DNA viruses and is actively targeted for immune evasion (114, 115). Whilst a VACV inhibitor for IFI16 remains to be discovered, VACV encodes at least 2 molecules preventing DNA-PK-mediated sensing (39, 41).

## VACV Antagonists of DNA Sensing Responses

VACV employs several strategies to suppress cytosolic DNA sensing. The discovery of these viral inhibitors has served to enlighten these relatively recent innate signaling pathways and provide important insights into their mechanisms of activation and regulation. These inhibitors and strategies are reviewed below and summarized in Table 1 and Figure 1.

### Targeting DNA-PK (Proteins C16 and C4)

The first VACV protein reported to suppress cytosolic DNA sensing during infection was C16, first characterized by Fahy et al. (116). It is a 37.5 kDa non-essential protein comprising 331 amino acids, encoded by the *C16L* gene (WR010/209), two copies of which are present in the ITRs on both ends of the VACV genome. C16 is highly conserved among some OPVX members such as VARV, ECTV, and CPXV, while in others such as monkeypox virus (MPXV) or camelpox virus (CMLV) the sequence contains disruptions that alter the reading frame (116). Originally, C16 was found to enhance VACV virulence via an unknown mechanism (116). Only later was its role in DNA sensing inhibition revealed as a DNA-PK inhibitor. During VACV infection C16 binds to the Ku70/80 heterodimer of the DNA-PK complex using its C-terminal domain (41). This prevents the recognition of VACV DNA since the Ku proteins mediate DNA binding. An engineered VACV lacking C16 caused less weight loss and induced higher levels of cytokines and chemokines in mice after intranasal inoculation (41, 116). Furthermore, MVA, which contains a deletion of 5 amino acids in the Ku-binding domain, induced IFN-I production in a DNA-PK-dependent manner (88). It is worth noting that VACV C16 is a multifunctional protein that has also been shown to manipulate hypoxic signaling and reprogramme energy metabolism (117, 118).

More recently, a second VACV protein has been found to target DNA-PK. Protein C4 (WR024) shares 43% identity with C16; it is highly conserved between several members of the OPXV genus; and it is non-essential for virus growth (119). C4 exhibits a similar function to C16 in regards to DNA sensing inhibition (39). C4 uses its C-terminal region to bind the Ku proteins and this reduces IRF3 phosphorylation and cytokine induction. Indeed, the binding of both C16 and C4 to Ku70 has been narrowed to three conserved amino acids. Viruses lacking expression of each of these proteins were attenuated in an intranasal model of infection inducing enhanced recruitment of immune cells and T cell activation, but remained as virulent as their parental viruses after intradermal inoculation (116, 119). Interestingly, a double deletion virus lacking both C16 and C4 revealed attenuation upon intradermal injection, demonstrating that these proteins had redundant *in vivo* roles in this model of infection (39). Why VACV devotes three genes (two copies of C16L and a copy of C4L) to suppress DNA-PK cytosolic DNA sensing remains unknown, but an explanation might be the high abundance of DNA-PK, particularly in fibroblasts, a primary cell target in the skin. Interestingly, it has been recently shown that DNA-PK can activate a second DNA sensing pathway completely independent of STING activation (93). This could further emphasize the role of C16 and C4 in immunomodulation and DNA sensing inhibition given that these proteins may also inhibit SIDSP.

### Targeting cGAS (Protein F17)

Besides the discovery of VACV inhibition of DNA-PK evidence existed that VACV was able to prevent STING activation by other mechanisms (94). One of these was reported by Meade et al. and involved protein F17 (42). F17 (WR056) is a conserved structural protein that constitutes the largest component of the virion lateral bodies and is essential for the formation of infectious virions (120–122). F17 exploits a complex cellular circuit connecting immune sensing and the metabolic rheostat mTOR (mammalian targets of rapamycin). mTOR is composed of two different complex systems, namely mTOR1 and mTOR2, both of which are regulated by distinct subunits conforming a negative feed-back loop (123, 124). F17 sequesters the subunits Raptor and Rictor so their regulatory feed-back is disrupted, allowing VACV to usurp mTOR control from the cell. This leads to overactive mTOR that enhances protein synthesis, but also suppresses innate activation at multiple levels including cGAS downregulation by a process that involves Akt (125) and dysregulation of STING vesicles in the ER (42). These effects suppress IRF3 translocation and trigger a potent suppression of ISG induction in both fibroblasts and macrophages, particularly late during infection in agreement with F17 late expression (42, 109). In the absence of F17 VACV infection leads to detectable ISG expression that depend on cGAS, but not IFI16, presence (109), thus supporting the primordial role of cGAS as a poxvirus DNA sensor. Of note, cGAS deletion did not completely abrogate ISG responses in fibroblasts (109), making room for a second IRF3-activating pathway sensing VACV in these cells. The high conservation of F17 not only across OPXV, but also across most vertebrate poxviruses, highlights the fundamental functions of this protein in poxvirus infections.

### Targeting cGAMP (Protein B2)

cGAMP plays a pivotal role in antiviral DNA sensing responses. As a product of activated cGAS it binds STING and triggers innate immune activation in the infected cell. However, it also induces antiviral responses in neighboring cells by multiple mechanisms. cGAMP is transferred to neighboring cells via gap junctions (61) and membrane fusion events (126), is incorporated into exiting viral particles (127, 128) and imported into cells from the extracellular milieu via the transporter SLC19A1 (62, 63). Via these mechanisms cGAMP has the capacity to activate non-infected cells including immune cells and constitutes a potent antiviral signaling molecule. VACV neutralizes the effects of cGAMP by encoding a cGAMP nuclease in gene *B2R* (WR184), discovered by Eaglesham et al. and named poxvirus immune nuclease (poxin) (36). Poxin was identified in a screen for viruses able to destroy cGAMP, which included 24 viruses belonging to 13 different viral families. Only VACV was found to degrade cGAMP, a reflection of its unique nature as a cytosolic replicating virus, and mass spectrometry confirmed the activity to derive from product B2. Poxin binds and linearises cGAMP cleaving the 3′ -5′ bond and converting it into linear Gp[2′ -5′] Ap[3′]. VACV lacking Poxin shows no defect in growth, but displays a significant reduction of viral titer in mice (36). Homologs of Poxin are found in baculovirus and their insect hosts, perhaps revealing a common origin since insect poxviruses and baculoviruses share ecological niches. Within the *Poxviridae*, Poxin is not universally conserved and surprisingly in most OPXV it appears as vSchlafen, a fusion of Poxin with a second protein with high similarity to the mammalian family of Schlafen proteins (also known as gene *B3R* in VACV). Deletion of the entire vSchlafen or only its cGAMP nuclease domain in the context of ECTV renders the virus unable to suppress IRF3 activation during infection and leads to a dramatic 5-log drop in virulence in mice (99), the natural host of ECTV. Despite its critical role in counteracting the antiviral effects of cGAMP both *B2R* and *B3R* are swarmed with inactivating mutations in VARV. Poxin and vSchlafen are early genes, a class of genes expressed from within intact cytoplasmic viral cores (8–10). This allows production of early immune evasion factors before the viral genomic DNA is released and exposed to DNA sensors, and in conjunction with late factors like F17, it ensures complete suppression of innate activation throughout the entire life cycle.

### Downstream IRF3 Inhibitors

Beyond the aforementioned, VACV possesses several different non-essential immunomodulatory proteins, most of which were reported when our knowledge on cytosolic DNA sensing was in its infancy. The discovery of DNA sensing pathways provides now a new dimension to their seemingly redundant roles. In most cases the function of these molecules as DNA sensing modulators has not been formally proven, but when the molecular mechanism of action has been elucidated, their antagonistic role can be anticipated. VACV proteins acting downstream of STING, and therefore expected to antagonize DNA sensing, include protein C6, which acts at the level of TBK1/IKKε. C6 binds to the cellular proteins TANK, NF-κB-activating protein (NAP)-1 and similar to NAP1 TBK1 adaptor (SINTBAD) (40). All three molecules work as adaptors for TBK1 and share a conserved TBK1/IKKε-binding domain (129). TBK1 and its adaptors act as a convergence point for TLRs, RNA and DNA sensing pathways and consistent with this, C6 is able to suppress IFN-I induction in response to poly(I:C), poly(dA-dT) and RNA virus infection, but did not affect NF-κB activation. VACV lacking C6 expression replicated normally in cell culture, but was significantly attenuated in mice (40). C6 was also shown to suppress IFN-I signaling (130, 131). Given this multifunctionality C6 becomes an important VACV IFN antagonist and its deletion increases immunogenicity in mice both in the context of virulent and avirulent VACV such as MVA where C6 is not inactivated (132–136). The related VACV protein K7 also targets the TBK1/IKKε complex to inhibit IRF3 activation. As well as possessing the ability to inhibit NF-κB activation (137), K7 binds to and inhibits the DEAD-box RNA helicase DDX3, a TBK1 adaptor and substrate required for optimal IRF responses (44, 138). K7 binds to the N-terminal region of DDX3 to inhibit its function, ensuring a decreased TBK1/IKKε-dependent IFN-β promoter induction (44, 139). Like C6, K7 contributes to VACV virulence in mice and its removal leads to enhanced immunogenicity and memory immune responses (132, 134). Finally, the VACV Bcl-2 protein N2 acts as an IRF3 inhibitor in the nucleus, although its specific target remains elusive (47). N2 is dispensable for virus growth, but contributes to virulence (47), and MVA deleted for gene N2L enhances immunogenicity and the immune response to infection, marked by an increase in the production INF-β and other pro-inflammatory cytokines immune activation (140).

### Downstream NF-κB Inhibitors

Multiple studies have demonstrated that production of inflammatory cytokines via STING-dependent DNA sensing requires NF-κB responses that depend on the IKK complex and the NF-κB heterodimer. VACV encodes multiple immunomodulators known to suppress NF-κB activation and detailed molecular mechanisms of action exist for several of them (16). VACV protein B14 is a Bcl-2-like protein encoded by the VACV *B14R* gene that is well-conserved among OPXV such as VARV and CPXV and contributes to virulence (37, 141). B14 binds to the N-terminal kinase domain and the scaffolding and dimerisation domain of IKKβ, and prevents its phosphorylation and activation, which in turn prevents the phosphorylation and subsequent degradation of IκBα (38, 142). This interaction is mediated by a surface of B14 that is otherwise utilized for dimerisation (37, 143). B14 can also activate the mitogen-activated protein kinase (MAPK)/activator protein 1 (AP-1) pathway (144) and is inactivated in MVA (145), confirming its non-essential role in virus growth. The related VACV protein N1 is a potent virulence factor that serves a dual role as inhibitor of apoptosis and inflammatory signaling (37, 45, 46, 107, 146, 147). Both functions map to different binding interfaces of the protein (45) and the ability to block NF-κB activation correlates with an impaired CD8 T cell effector and memory response (148). Although its exact mechanism of action for suppressing innate immune activation remains unknown, the inhibitory action of N1 on NF-κB signaling is believed to be downstream of the TRAFs. N1 is also known to be modified by ubiquitylation during infection, but this did not affect its ability to suppress NF-κB activation (149). A third VACV Bcl-2 protein evolved to suppress NF-κB signaling is A49, which contains an N-terminal region that mimics the IκBα degron sequence that mediates its proteasomal destruction (33, 150). A49 is phosphorylated by IKKβ and subsequently recognized by the E3 ubiquitin ligase β-TrCP, but unlike IκBα it is spared because it lacks the ubiquitin acceptor sites located upstream of the degron (33, 150). Using this mechanism A49 binds tightly to β-TrCP only when IKKβ is activated and canonical β-TrCP targets including IκBα and β-catenin accumulate in their phosphorylated forms and are not processed (33, 150, 151). A49 antagonism of NF-κB contributes to virulence (33, 34), but mutant A49 viruses unable to bind β-TrCP retain some degree of virulence suggesting the existence of other β-TrCP-independent functions, perhaps mediated by a second product identified in the A49 ORF (34, 152). In addition to these proteins, several VACV proteins including K1 and A55 have been shown to act at the level of the NF-κB heterodimer preventing their translocation or their normal processing (35, 43, 153). Thus, they also have the potential to suppress STING-induced NF-κB activation. Lastly, evidence exists that VACV downregulates NF-κB-dependent gene expression after p65 translocation by yet unidentified viral strategies (154).

## Implications for the Therapeutic Use of VACV

VACV-based therapeutics involve the use of VACV as a vaccine vector and oncolytic agent, as well as the use of VACV-derived proteins and peptides as biologicals. The development of VACV as a vaccine and for virotherapy holds promise and some forms of the virus are now in clinical studies, whereas the development of VACV-derived biologicals is at a much less advanced stage. VACV strain ACAM2000 (a derivative of Wyeth's Dryvax vaccine) and MVA (marketed as Jynneos) are approved by the USA Food and Drug Administration (FDA) for their use against smallpox and monkeypox, and various VACV strains have been engineered to carry heterologous antigens for diseases such as AIDS, malaria or tuberculosis amongst others (2). Enhancing immunogenicity and/or attenuating the virus remain desirable goals to increase VACV safety profile, elicit stronger immunological memory and reduce dosage and administration regimes. The discovery of DNA sensing pathways and their critical biological roles creates novel opportunities for the improvement of VACV as a therapeutic agent. The recognition of foreign incoming DNA in the cytosol triggers a potent immune and inflammatory reaction that includes IFN-I and IFN-III responses known to be beneficial for immune activation. Therefore, strategies aimed at increasing recognition of the VACV genome and boost intracellular DNA sensing signaling are likely to enhance vaccine efficacy and immunogenicity. These may include the deletion of immunomodulatory virulence factors from the virus or the co-administration of STING agonists. Evidence exists for the enhancement of immunogenicity and memory immunity upon removal of VACV immunomodulators [recently reviewed in (155)]. Some of these immunomodulators are listed above as viral DNA sensing antagonists. Therefore, this strategy seems logical to enhance IFN and cytokine production. An important aspect of consideration is whether removal of these viral immunomodulatory proteins is expected to impact on cGAMP levels. cGAMP triggers a STING-dependent, but cGAS-independent induction of cytokines when transferred between cells. Horizontal transfer of cGAMP during infection therefore has the potential to boost and shape the adaptive response. Indeed, deletion of ECTV cGAMP nuclease vSchlafen led to a marked IFN-I signature in the draining lymph node and spleen that correlated with enhanced NK cell activation and survival to an otherwise lethal infection (99). Furthermore, studies have reported how the cGAS-STING axis promotes the generation of cytotoxic T cells (CTL) via the expression of IFN-I and the cross-presentation of antigens by dendritic cells (DC), in some cases after administration of exogenous cGAMP (156–159). Therefore, preventing the degradation of cGAMP by, for instance, removal of Poxin/B2 from VACV is likely to contribute favorably to the outcome of vaccination. A similar beneficial outcome may derive from the removal of VACV DNA-PK antagonists so DNA-PK-mediated innate immunity responses are unleashed.

A second positive prospect from these studies is the value of STING agonists as adjuvants and anti-virals. Although smallpox was eradicated 40 years ago, the importance of compounds with anti-OPXV activity is increasing due to the emergence of zoonotic OPXV infections, particularly by MPXV. MPXV causes human monkeypox, an emerging zoonotic smallpox-like disease that has a mortality rate of 5–10% and has caused several outbreaks with hundreds of cases in Central and West Africa and outbound travelers (160, 161). At present, ST-246 (marketed as tecovirimat) is the only FDA-approved drug for the treatment of OPXV infections and although it has good activity range against multiple OPXV species including VARV (162–164), alternative strategies are still desirable. Administration of exogenous cGAMP has been shown to elicit strong immune responses and protect mice against lethal ECTV infection (98, 99). This suggests that STING immunity is an attractive antiviral therapeutic target, although further experimentation is needed to determine the effects and specificity of cGAMP delivery pre- and post-infection as well as through different routes of inoculation.

A similar beneficial outcome in enhancing cGAS-STING signaling by either removal of viral immunosuppressive strategies or delivery of STING agonists might be expected in the context of oncolytic VACV. Oncolytic viruses are those that can selectively infect and/or grow in tumor cells leading to their destruction. VACV is a popular oncolytic virus for multiple reasons including its wide cell tropism and cytotoxicity, its ability to grow in hypoxic environments, the possibility for stable transgene expression and the fact that it does not integrate its DNA in the genome of the cell (165–167). Like tumor cells, VACV exploits multiple strategies to suppress cell death (168), some of which might be redundant with cancerous cells. However, whereas cancers tend to establish an immunosuppressive tumor microenvironment, VACV is immunogenic and is capable of altering the immune landscape, co-stimulating acquired anti-tumor immunity following replication within tumor tissues. In the case of immunogenic tumors, it has been reported that their recognition by the host immune system relies on STING-dependent cytosolic DNA sensing (156, 159). It has also been shown that intratumoral injection of MVA generated adaptive anti-tumor immunity in melanoma and colon cancer that was dependent on STING (169) and a GM-CSF (granulocyte-macrophage colony-stimulating factor)-secreting vaccine showed increased anti-tumor efficacy when formulated with STING CDN agonists (170). In addition, susceptibility of certain cancers to viral oncolysis correlates with STING and STING signaling (171, 172). Collectively, these studies demonstrate the importance of the cGAS-cGAMP-STING axis in immunotherapy and offer scope for the improvement of oncolytic VACV and its therapeutic potential.

Finally, a number of human diseases have been connected with cytosolic DNA sensing. Mutations in several human genes result in the accumulation and mislocalisation of DNA molecules leading cGAS activation, and this is best exemplified in the form of the Aicardi-Goutières syndrome (AGS), a rare devastating disease characterized by systemic inflammation (173). The persistent stimulation of cGAS-STING signaling has also recently been associated with systemic lupus erythematosus (SLE) (174), a much more prevalent disease associated with IFN-I dysregulation. Our increased knowledge on VACV antagonism of cytosolic DNA sensing may identify novel components or regulatory mechanisms of these cellular pathways and may reveal novel strategies to counteract the functions of these attractive therapeutic targets. For instance, detailed mechanistic and structural insights into VACV DNA sensing antagonists can allow the development of small molecule inhibitors mimicking the mode of action of the viral proteins. A proof of principle for these approaches is provided by the design of peptides deriving from VACV TLR inhibitors A46 and A52 (175, 176).

## Conclusions

As our knowledge on nucleic acid immunity and inflammation expands it is becoming increasingly clear that the activation and regulation of intracellular DNA sensing has broad implications for human health and disease. VACV is a fantastic tool for discovery in human biology and virus pathogenesis as well as an important therapeutic tool. VACV has already proven pivotal in the discovery of cellular mechanisms for regulation of DNA sensing including uncovering the existence of viral and cellular cGAMP nucleases. VACV targets innate immune signaling at multiple levels and given the importance of DNA sensing for a cytosolic replicating virus it is likely that new viral inhibitors remain to be identified. Further research is also needed to address how VACV is sensed as the virion uncloaks and is recognized by cellular sensors. The identity and relative importance of these in the different cell types that are relevant for VACV infection and spread is also a necessary area of investigation. These findings need to be reciprocated in other poxviruses and can enlighten similar processes in viruses with similar biology such as the African Swine Fever virus and intracellular bacteria. Furthermore, many questions remain unanswered about how cells launch cell intrinsic defense mechanisms against VACV that involve recognition of its genome. Importantly, this knowledge is crucial to increasing the potential of VACV-based therapeutics both in the form of vaccines and oncolytic virus.

## Author Contributions

Conceptualization and funding acquisition: CM. Writing: ME-J, MT, and CM. All authors contributed to the article and approved the submitted version.

## Conflict of Interest

The authors declare that the research was conducted in the absence of any commercial or financial relationships that could be construed as a potential conflict of interest.
